# Supplementary material for: Role of diversity-generating retroelements for regulatory pathway tuning in cyanobacteria
Source: BMC Genomics. 2020 Sep 25;21:664. doi: 10.1186/s12864-020-07052-5 (PMC7517822; doi:10.1186/s12864-020-07052-5)
Supplement: Supplementary file 6 — Additional file 6 Figure S1. Hanks-type Kinase Motif Characterization in VPs. Alignment of known “Hanks and Hunter-type” (S/T) kinase domains to the kinase domains of all DGR-VPs, Remote VPs, and VP Paralogs from this dataset. Motifs I-XI highlighted in blue. The top eight sequences denoted “STKII” are known Type II S/T kinases from Zhang et al. 2007 [14]. [file 12864_2020_7052_MOESM6_ESM.pdf]

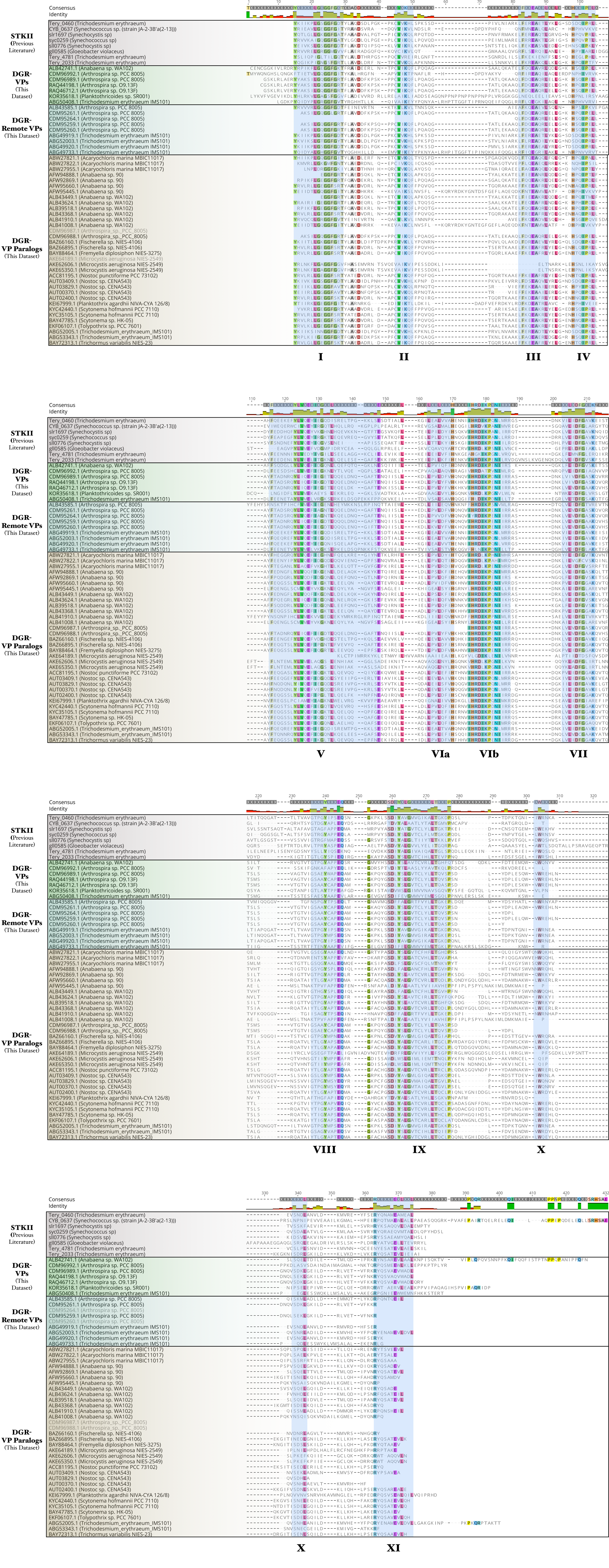
Consensus
Identity
STKII
(Previous Literature)
DGR-VPs
(This Dataset)
DGR-Remote VPs
(This Dataset)
DGR-VP Paralogs
(This Dataset)
I
II
III
IV
V
VIa
VIb
VII
VIII
IX
X
X
XI
